# Retraction Note: Mechanically interlocked architecture aids an ultra-stiff and ultra-hard elastically bendable cocrystal

**DOI:** 10.1038/s41467-021-25762-6

**Published:** 2021-10-20

**Authors:** Somnath Dey, Susobhan Das, Surojit Bhunia, Rituparno Chowdhury, Amit Mondal, Biswajit Bhattacharya, Ramesh Devarapalli, Nobuhiro Yasuda, Taro Moriwaki, Kapil Mandal, Goutam Dev Mukherjee, C. Malla Reddy

**Affiliations:** 1 Department of Chemical Sciences, Indian Institute of Science Education and Research (IISER) Kolkata, Mohanpur, West Bengal 741246 India; 2 Center for Advanced Functional Materials, Indian Institute of Science Education and Research (IISER) Kolkata, Mohanpur, West Bengal 741246 India; 3 Japan Synchrotron Radiation Research Institute (JASRI), 1-1-1 Kouto, Sayo, Hyogo, 679-5198 Japan; 4 Department of Physical Sciences, Indian Institute of Science Education and Research (IISER) Kolkata, Mohanpur, West Bengal 741246 India

**Keywords:** Mechanical properties, Mechanical properties, Structural properties

Retraction to: *Nature Communications* 10.1038/s41467-019-11657-0, published online 19 August 2019.

The authors wish to retract this paper after they were notified about possible artefacts in their data. The authors re-analysed the face indexing on the crystal and agree that the longest dimension of the crystal is along the stacking direction parallel c-axis in agreement with^1^. Furthermore, the authors agree that the accurate description of atoms using isotropic ADPs is more appropriate than achieving anisotropic ADPs considering the limitations with the data from bent crystals. This impacts the result on the elastic bending mechanism and the re-evaluated data confirm that the observed changes in c-axis are in agreement with the expected compression on the interior and expansion on the exterior of the crystal according to elastic beam bending theory^1,2^. The revised face indexing suggests that the interlocked tapes which are stacked along the c-axis are likely to be less effective in obstructing the shear deformation of molecules during nanoindentation experiments. Based on this finding, the authors are unable to explain the high elastic modulus and high hardness values of the crystal. Considering the statistical significance, the authors think that the high values could be due to instrument compliance issues or reasons which are not yet clear.
